# Application of a multiplex CRISPR/Cas9 strategy for elimination of selection markers from transgenic plants

**DOI:** 10.3389/fgeed.2025.1633104

**Published:** 2025-09-03

**Authors:** Mohammed Rafi, Mohamed ElSiddig, Maitha Aldarmaki, Mariam Al Nuaimi, Suja George, Khaled M. A. Amiri

**Affiliations:** ^1^ Khalifa Center for Genetic Engineering and Biotechnology, Al Ain, United Arab Emirates; ^2^ Department of Biology, College of Science, United Arab Emirates University, Al Ain, United Arab Emirates

**Keywords:** CRISPR/Cas9, marker-free transgenic, polycistronic tRNA-gRNA, selection marker gene, tobacco

## Abstract

Selectable marker genes (SMGs) are essential for identifying transgenic plants but raise concerns regarding biosafety, regulatory compliance, and public acceptance. In this study, we used a CRISPR/Cas9-based strategy to eliminate the SMG from transgenic tobacco plants. Leaf discs from plants carrying DsRED (SMG) and aminoglycoside phosphotransferase (gene of interest, GOI) were re-transformed with a CRISPR vector containing four gRNAs designed to target both flanking regions of the SMG cassette. Approximately 20% of the regenerated shoots exhibited loss of red fluorescence, and PCR and sequencing analyses confirmed that about half of these carried a smaller amplicon, indicating a successful SMG excision efficiency of around 10%. Mutation analysis further revealed the presence of small indels at gRNA target sites, in addition to the deletion of SMG cassette. Quantitative real-time PCR (qPCR) analysis confirmed the absence of DsRED expression in SMG-deleted lines, while the Cas9 and GOI remained actively expressed. The SMG-free plants displayed normal growth, flowering, and seed production, indicating CRISPR marker excision had no adverse effects on plant development and fertility. In addition, Cas9-free, marker-free transgenic plants were recovered through segregation in T1 generation. This approach is adaptable to various transgenic plant species and provides a practical solution for generating marker-free transgenic crops, thereby enhancing their acceptance and commercialization.

## Introduction

Genetic engineering of crops with foreign genetic material is a widely used technique to develop novel varieties with improved traits and desirable characteristics (Kumar et al., 2020; Kamthan et al., 2016; Mackelprang and Lemaux, 2020). Genetic transformation techniques, whether utilizing *Agrobacterium tumefaciens*-mediated methods or direct gene transfer approaches, commonly rely on selectable marker genes (SMGs) to aid in identifying and selecting transgenic material (Ziemienowicz, 2001; Miki and McHugh, 2004; Súnico et al., 2024). Once transgenic plants carrying the gene of interest are regenerated and characterized, selectable marker genes (SMGs) are no longer necessary. However, their continued presence in the final plant product raises significant environmental and biological safety concerns (Teng, 2008). The possibility of horizontal gene transfer and the introgression of marker genes into weedy relatives, non-transgenic crops, and pathogens is a concern for many (Teng, 2008; Turnbull et al., 2021). Additionally, public acceptance of genetically modified (GM) crops is hindered by health-related concerns associated with the persistent presence of SMGs (Halford, 2019; Aldemita et al., 2015).

The commercial release of transgenic plants containing antibiotic marker genes is strictly regulated in several countries and faces opposition from numerous non-governmental organizations (NGOs), industries, and regulatory bodies (Singh et al., 2022). Unlike gene-edited plants, where foreign genetic material can be removed in subsequent generations, transgenic plants are subject to strict regulations in countries that permit their commercialization (Gu et al., 2021). These regulatory hurdles significantly delay their release and limit their acceptance. From a metabolic point of view, including an additional gene that doesn’t contribute to the desired trait development poses the potential concern of metabolic drain (Li et al., 2009). Additionally, only a handful of SMGs are regularly used in the development of transgenic plants and this limits the process of gene stacking through re-transformation (Zhao et al., 2019). Hence, developing marker-free transgenic plants would be very advantageous to their eventual commercial release.

Several strategies such as co-transformation, site-specific recombination, and transposon-mediated approaches, have been developed to eliminate SMGs from transgenic plants with varying degrees of success (Woo et al., 2015; Tuteja et al., 2012). In the co-transformation method, two separate T-DNAs—one harboring the SMG and the other containing the gene of interest (GOI)—are introduced into plant cells simultaneously. Marker-free lines are subsequently identified among the progeny based on the genetic segregation of the two T-DNAs (Ling et al., 2016). The Cre/lox system offers another widely used method, where the SMG is flanked by loxP sites and removed post-transformation via recombination, leaving the GOI intact in the plant genome (Bai et al., 2008; Kleidon et al., 2020; Nandy et al., 2015). Additionally, transposon-based systems such as the Activator/Dissociation (Ac/Ds) elements from maize have been employed. In this approach, the Ds element carrying the GOI, along with Ac transposase and the SMG, are co-delivered into plant cells. The Ac transposase mediates excision and reintegration of the Ds element at a new genomic location, and following segregation, transgenic plants can be recovered that retain only the GOI and lack both the SMG and original T-DNA insertion (Lazarow et al., 2013; Gao et al., 2015).

Genome editing in plants using CRISPR/Cas (Clustered Regularly Interspaced Short Palindromic Repeats)/(CRISPR-associated protein) system has revolutionized modern plant biotechnology by enabling precise modifications of the plant genome (Zhu et al., 2020; Cardi et al., 2023). This technology employs a Cas9 endonuclease, guided by short guide RNAs (gRNAs), to introduce targeted double-strand breaks at specific genomic loci. These breaks are repaired by the plant’s natural DNA repair mechanisms, such as non-homologous end joining (NHEJ) or homology-directed repair (HDR), leading to insertions, deletions, or targeted substitutions (Zhu et al., 2020; Cardi et al., 2023). One of the key advantages of this technology lies in its ability to produce transgene-free mutants through the segregation of the transgene and edited loci in subsequent generations, provided these loci are unlinked (Bhattacharjee et al., 2023; Ricroch et al., 2017). Unlike other methods, CRISPR/Cas editing does not leave any foreign DNA at the excision site, allowing for the complete removal of introduced genetic material in subsequent generations.

In recent years, a few CRISPR-based strategies have been developed specifically to obtain marker-free transgenic plants. One approach involves placing both the SMG and the Cas9 cassette between gRNA target sites within a single T-DNA, enabling their self-removal following transformation (Hu and Yu, 2022). In another strategy, CRISPR/Cas9-based HDR was utilized for efficient removal of selectable marker genes (Tan et al., 2022).

We developed a multiplex CRISPR/Cas9-based vector system capable of precisely removing SMGs from previously transformed and established transgenic lines. This system involves introducing a CRISPR/Cas9 vector carrying multiple gRNAs designed to target regions flanking the SMG cassette in the transgenic plant, enabling the induction of large deletions encompassing the entire cassette. Previous reports have shown that the use of multiple gRNAs enhances the frequency of large fragment deletions through NHEJ (Ordon et al., 2023; Jin and Marquardt, 2020; Zhou et al., 2014). In our system, the high-efficiency deletion of the SMG in the T0 generation, followed by segregation of the CRISPR components in T1, facilitates the recovery of marker-free and Cas9-free transgenic plants.

Unlike earlier CRISPR-based strategies that introduce the GOI, SMG, and CRISPR components simultaneously—followed by removal of the SMG and editing machinery—our method offers an efficient solution for the targeted elimination of antibiotic marker genes from already established transgenic plant lines, particularly those intended for commercial release. We have demonstrated the method in model plant tobacco, but, assessing the efficiency of marker gene elimination for the specific species and genotype of interest is critical. Transgenic plants can be developed using existing techniques, enabling the evaluation of multiple lines to identify the most promising variety. However, as the SMG only needs to be removed from a single, high-performing transgenic line designated for release, the method remains highly practical and effective even if the overall percentage of complete elimination of SMG is below 10%.

## Materials and methods

### Plant material and growth conditions

*Nicotiana tabacum* L. cv. Petit Havana SR1 wild-type seeds were sterilized by immersing them in 70% ethanol for 1 min, followed by a 20-min treatment with 10% bleach. After sterilization, the seeds were thoroughly rinsed with sterile distilled water to remove any residual bleach. They were then plated on germination medium composed of half-strength Murashige and Skoog (MS) basal medium with vitamins, supplemented with 1% sucrose and 0.7% plant agar, and adjusted to a pH of 5.8. The plates were incubated under controlled conditions at 22°C with a 16-h light/8-h dark photoperiod to promote germination and seedling growth.

### Binary vector design and cloning

All primers utilized in this study are listed in Supplementary Table S1, and the vector maps are presented in Supplementary Figure S1. The plant transformation vector pRI 201-AN, obtained from Takara Biosciences (www.takarabio.com), contains the aminoglycoside phosphotransferase gene, which confers kanamycin resistance as an antibiotic selection marker. The fluorescent reporter gene DsRED was amplified from plasmid pEGB 35S:DsRED:Tnos (GB0361) (www.addgene.org, Plasmid #68220) using primers RED_FRAG_F and RED_FRAG_R. The vector pRI 201-AN was linearized by digestion with the restriction enzymes NdeI and SalI (NEB). The amplified DsRED gene was inserted into the linearized vector using In-Fusion cloning, following the manufacturer’s instructions (Takara Biosciences, www.takarabio.com). Successful cloning was confirmed by PCR and sequencing with primers Gene1_SEQ_F and Gene1_SEQ_R. The resulting recombinant vector was designated as pRED-AN.

### Agrobacterium-mediated wild-type (WT) tobacco transformation

Eight-week-old wild-type (WT) tobacco seedlings were used for transformation. The recombinant vector pRED-AN was introduced into *Agrobacterium tumefaciens* strain LBA4404 using the freeze-thaw method (Chen et al., 1994). Leaf explants tobacco seedlings were transformed as described before (Clemente, 2006). Transgenic shoots were selected on a shoot regeneration medium (3% MS media + 2 mg/L Kinetin + 1 mg/L IAA) containing 100 mg/L kanamycin and then verified by PCR and the observation of red fluorescence. The rooted plants were grown in soil and seeds were collected from a single transgenic line. A single T1 seedling from this line was subsequently used for transformation with the CRISPR vector to delete the SMG.

### Guide RNA design and CRISPR vector construction

**FIGURE 1:**
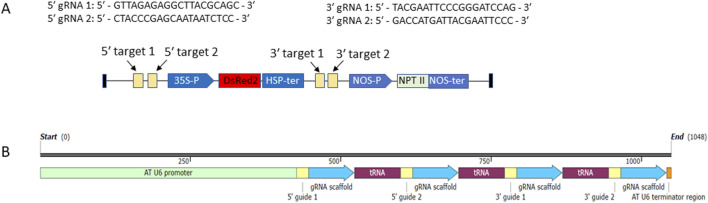
**(A)** T-DNA region of pRED-AN. SMG (DsRED) and GOI (aminoglycoside phosphotransferase, NPT II) cassettes are shown. The four gRNA target regions are marked. The sequences of the gRNAs are shown. **(B)** Structure of polycistronic tRNA-gRNA gene (PTG). The PTG expression is driven by Arabidopsis Pol III promoter At-U6. The positions of the four gRNAs, the gRNA scaffolds and intervening tRNA sequences are marked. The transcription is terminated by Arabidopsis At-U6 terminator (TTT).

In this study, DsRED was used as the selection marker gene (SMG), while the aminoglycoside phosphotransferase gene, which confers tolerance to kanamycin, was treated as the gene of interest (GOI). gRNAs were designed using CRISPOR (https://crispor.gi.ucsc.edu/), with the *Nicotiana tabacum* reference genome (version GCF_000715135.1) serving as the off-target database for scoring each gRNA. Four gRNAs with minimal off-target scores were selected—two targeting the region 5′ of the SMG cassette (35S promoter–DsRED–HSP terminator) and two targeting the region 3′ of the SMG cassette (Figure 1A).

CRISPR/cas9 vector pHSE401 harboring Arabidopsis U6-26 promoter for expression of gRNA, Cas9 endonuclease from the *Streptococcus pyogenes* and hygromycin B phosphotransferase gene as antibiotic selection marker for selection of transgenic plants was obtained from addgene (www.addgene.org, Plasmid #62201). To use the endogenous tRNA system for simultaneous production of the four gRNAs from a single promoter, a polycistronic tRNA-gRNA (PTG) gene was designed as described previously (Xie et al., 2015) (Figure 1B). A 153-nt segment consisting of the gRNA scaffold sequence followed by tRNA sequence (Xie et al., 2015) was synthesized by the manufacturer (Macrogen, Seoul, Korea) (Supplementary Figure S2). gRNAs were incorporated into this DNA segment in front of the scaffold by amplification with specific primers. The four PCR fragments each carrying a gRNA-scaffold-tRNA segment were directionally assembled by NEBuilder® HiFi DNA Assembly following manufacturer’s instructions (www.neb.com). The assembled fragment (Supplementary Figure S2) was cloned into BsaI digested pHSE401 and the cloning was confirmed by PCR and sequencing with primers AT_U6_F and AT_U6_R. The resulting recombinant vector was designated as pHSE401-SMGDEL.

### Transformation of transgenic tobacco and screening of CRISPR mutants

Leaf discs from pRED-AN T1 transgenic plant were transformed with pHSE401-SMGDEL as described in the previous section. Transgenic shoots were selected on a shoot regeneration medium containing 25 mg/L hygromycin. 200 regenerating shoots were analyzed for the presence of red fluorescence and those displaying no red fluorescence were transferred for rooting and genotyping. Genomic DNA was extracted from young leaves of these plants using an in-house protocol. The existence of pRED-AN T-DNA and pHSE401-SMGDEL T-DNA was confirmed by PCR using primers specific to genes conferring tolerance to kanamycin and hygromycin respectively (Supplementary Table S1). To analyze SMG cassette deletion, regions flanking the 4 target sites were amplified with specific primers pRED-AN_F and pRED-AN_R and analyzed by electrophoresis. These amplicons were then purified using the Qiagen QIAquick PCR Purification Kit (Hilden, Germany) and sequenced by Sanger sequencing. Microscopic images for RFP expression were captured with a Leica Thunder Imager System (Leica Microsystems Ltd., Switzerland) and analyzed using LAS X software.

### Analysis of mutations

For identifying patterns of mutation, PCR amplicons of SMG cassette were purified using the Qiagen QIAquick Gel Extraction Kit (Hilden, Germany). The purified PCR fragments were cloned into plasmid using the Zero Blunt™ TOPO™ PCR Cloning Kit (www.thermofisher.com) according to the manufacturer’s instructions, and positive colonies were selected by colony PCR with primers pRED-AN_F and pRED-AN_R flanking the target regions. Plasmids were extracted from at least five positive clones and sequenced using the same primers at Macrogen Inc (Seoul, Korea). SnapGene (www.snapgene.com) software was used to identify mutations.

### qPCR analysis

Total RNA was extracted using TRIzol reagent (ThermoFisher Scientific, Cat # 15596026) from two pHSE401-SMGDEL transgenic lines positive for all three tested genes (Cas9, aminoglycoside phosphotransferase, and DsRED) and five lines positive for Cas9 and aminoglycoside phosphotransferase but negative for DsRED, based on in-house protocol. Expression levels of Cas9 endonuclease, aminoglycoside phosphotransferase and DsRED in these lines were verified by qRT-PCR. RNA reverse transcription was performed using QuantiTect Reverse Transcription Kit (Qiagen). Gene-specific primers were designed by Primer 3.0 software (Supplementary Table S1). The qRT-PCR reaction mixture was prepared using PowerUp™ SYBR™ Green Master Mix (Applied Biosystems™ by Thermo Fisher Scientific, Lithuania) and qRT-PCR profiling was performed using a fluorescence quantitative instrument (StepOnePlus™ Real-Time PCR System; Applied Biosystems™). Three biological replicates and three technical replicates were used for all qRT-PCRs. Tobacco actin gene (GenBank accession number: X69885.1) was used as internal reference (Schmidt and Delaney, 2010). The relative gene expression level was analyzed according to the 2^−ΔΔCT^ method (Livak and Schmittgen, 2001).

## Results

### Generation of pHSE401-SMGDEL transgenic tobacco plants and selection for SMG cassette deletion

**FIGURE 2:**
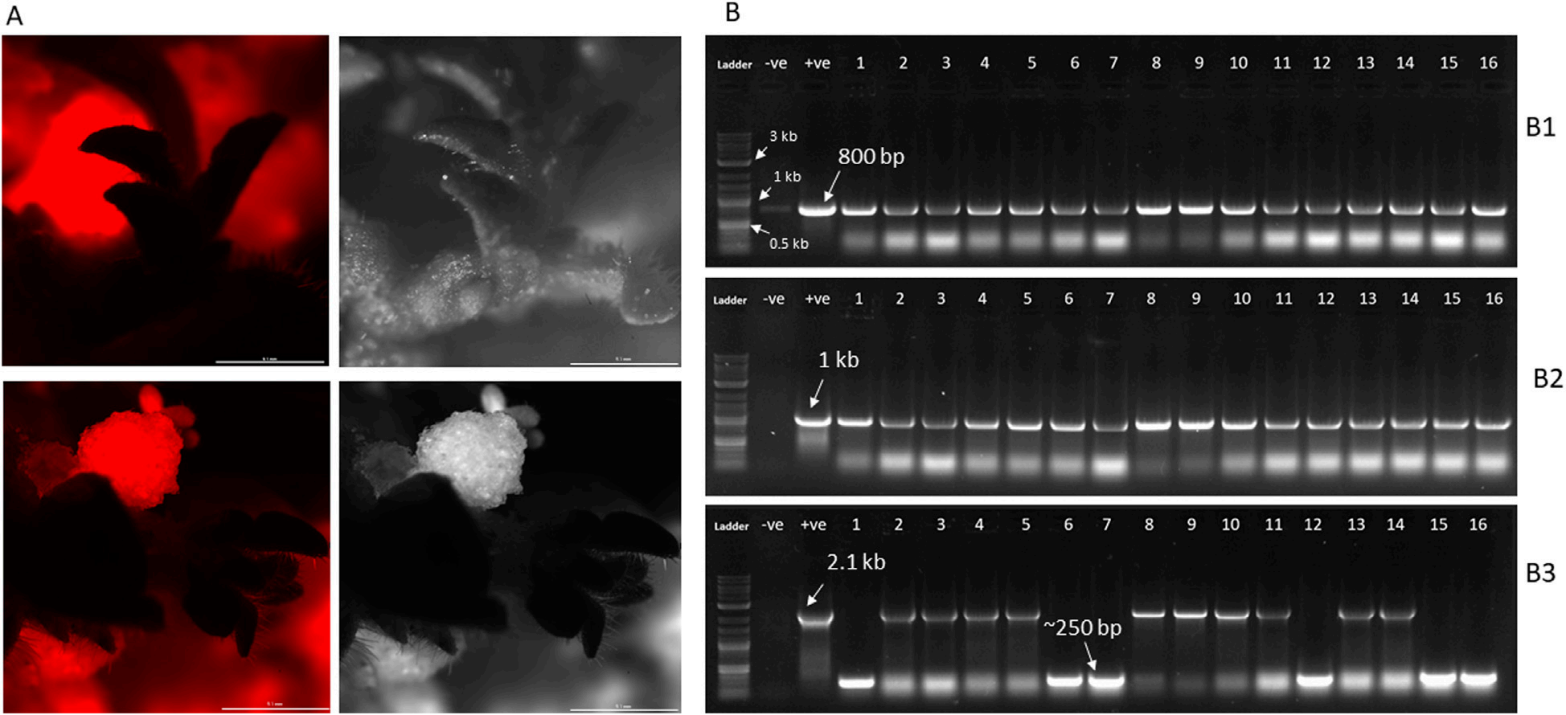
**(A)** Screening of pHSE401-SMGDEL transgenic lines for RFP expression. Fluorescent microscopic images of representative shoots lacking visible RFP is shown, along with bright field image. **(B)** PCR screening of pHSE401-SMGDEL transgenic lines lacking visible red fluorescence. 16 representative lines along with positive and negative controls are shown. **(B1)** PCR screening for the presence of aminoglycoside phosphotransferase gene (GOI), **(B2)** PCR screening for the presence of cas9 endonuclease, **(B3)** PCR screening for the presence or absence of 2.1 kb amplicon containing gRNA target regions and the SMG cassette.

To successfully eliminate the SMG cassette from transgenic plants, we designed a CRISPR vector (pHSE401-SMGDEL) carrying four gRNAs—two targeting the region upstream and two targeting the region downstream of the cassette. Seeds from a single line of T0 tobacco plants, transformed with pRED-AN and exhibiting high red fluorescence, were germinated on selection media containing 100 mg/L kanamycin. A single T1 seedling was selected and further grown. Leaf discs from this plant were used for transformation with the pHSE401-SMGDEL plasmid. A total of 200 well-formed shoots, aged 8 weeks and regenerated on media containing 25 mg/L hygromycin, were analyzed for red fluorescence. Of these, 80% displayed red fluorescence, while 20% did not, suggesting potential SMG cassette deletion (Figure 2A). The shoots lacking visible red fluorescence were rooted and grown further.

Forty of these plants were genotyped by PCR to check for the presence of the SMG cassette, CAS9 gene, and aminoglycoside phosphotransferase gene. All tested plants were PCR-positive for both the CAS9 gene, which is part of the pHSE401-SMGDEL T-DNA, and the aminoglycoside phosphotransferase gene, which is the gene of interest (GOI) from the original pRED-AN T-DNA (Figure 2B). When these plants were genotyped for the targeted SMG cassette deletion, 21 plants tested positive for the 2.1 kb cassette, indicating the presence of the SMG. It is possible that these 21 plants had low DsRED expression levels, leading to their initial visual selection as potential SMG-deleted plants. In contrast, 19 plants showed a much smaller band at around 250 bp, indicating complete deletion of the SMG cassette (Figure 2B).

### Analysis of mutations

Vector pHSE401-SMGDEL carries four gRNAs two each targeting either side of the SMG cassette in the pRED-AN T-DNA integrated into the pRED-AN transgenic tobacco plants. From the pHSE401-SMGDEL-transformed T0 plants lacking visible red fluorescence, 10 random plants from each group (those with the 2.1 kb band and those with the ∼250 bp band) were selected for mutation analysis.

**FIGURE 3:**
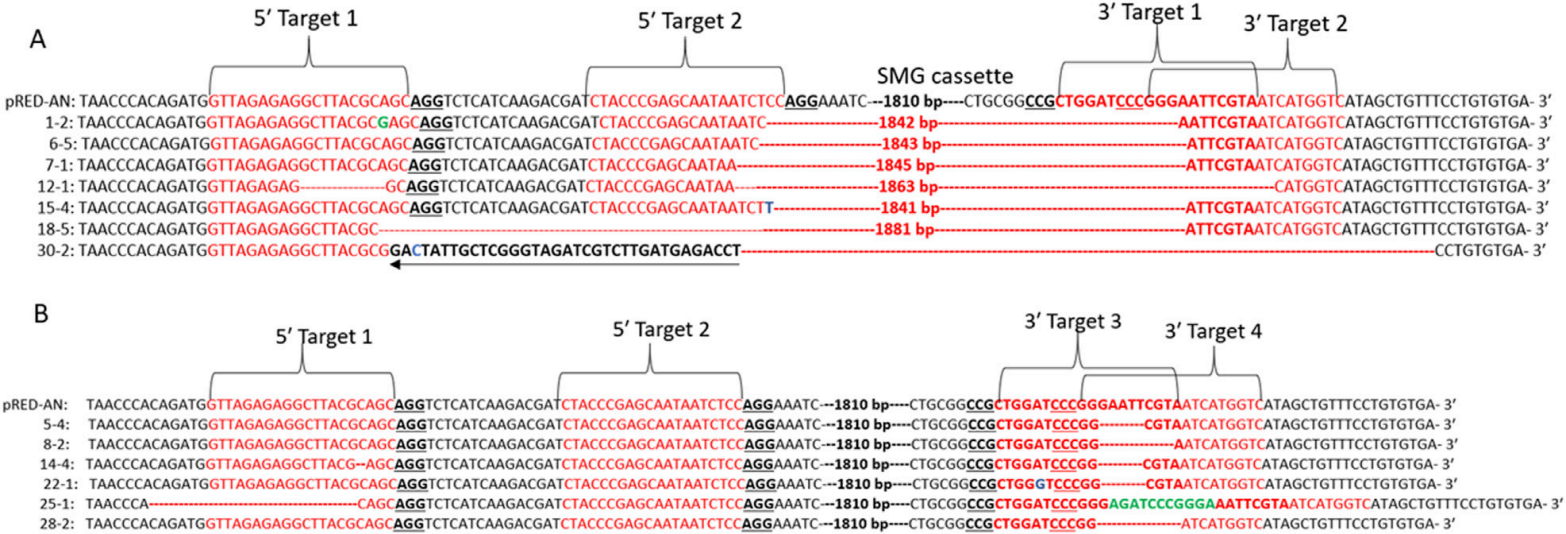
CRISPR/Cas9-based mutation and/or elimination of SMG from pRED-AN transgenic tobacco lines. **(A)** Genome-edited sequences of SMG cassette-deleted pRED-AN transgenic tobacco lines, **(B)** Genome-edited sequences of 2.1 kb PCR-positive lines. Sequences from unedited pRED-AN transgenic tobacco plants and representative edited lines are shown. The four target regions on either side of the SMG cassette are marked. The gRNA sequences are in red, and the PAM is underlined. Deletions are marked by dotted red lines, insertions are marked in green and substitutions are marked in blue. Inversion is underlined by black arrows. Only distinct patterns of mutations are shown.

To examine mutation patterns, the genomic region containing the target sites for the four gRNAs was amplified using flanking primers, cloned, and sequenced by Sanger sequencing. Sequencing of the PCR products from plants with the ∼250 bp amplicon confirmed complete deletion of the SMG cassette in all 10 sequenced plants (Figure 3A). Since 19 out of 200 regenerated shoots were PCR-positive for complete deletion, this indicates a nearly 10% frequency of successful SMG cassette elimination.

Large deletions, ranging from 1841 bp to 1881 bp were observed in the analyzed lines (Figure 3A). In addition to the large deletion leading to the elimination of the SMG cassette, small insertions, deletions and substitutions were also observed at different target sites. The target region of 5′ gRNA 2 was mutated in all lines analyzed where as only four out of ten lines showed mutations in the 5′ gRNA 1 target region. We couldn’t ascertain the mutation frequency of 3′ gRNA 1 in the SMG-deleted lines as the target regions for 3′ gRNA 1 and 2 overlapped. All analyzed lines showed mutations in 3′ gRNA 2 target region. One of the lines, line 30-2, showed inversion of the intervening regions between 5′ gRNA 1 and 2, in addition to the SMG cassette deletion (Figure 3A).

When the 2.1 kb amplicon from the other 10 plants were cloned and sequenced, eight out of ten plants showed mutations in at least one of the four target regions (Figure 3B). Surprisingly, target region of 5′ gRNA 2 which was mutated in all the SMG-deleted lines was not mutated in any of the 2.1 kb PCR-positive lines. 5′ gRNA 1 target showed deletions in two lines and 3′ gRNA 1 target showed a substitution in one of the lines. 3′ gRNA 2 target showed small deletions or insertions in all the lines analyzed (Figure 3B).

Although the plants positive for the 2.1 kb SMG cassette carried mutations, these mutations did not affect the expression of the DsRED gene, as they were outside the SMG cassette and did not impact the promoter or cause frameshifts in the DsRED coding sequence. It is possible that the 21 plants testing positive for the 2.1 kb SMG cassette had low DsRED expression levels, leading to their initial visual selection as potential SMG-deleted plants.

The ten SMG-deleted lines and ten 2.1 kb PCR-positive lines which were analyzed for mutation patterns were grown further in soil. All the plants grew normally and flowered and set seeds similarly to control un-transformed plants. The seeds from randomly selected SMG-deleted lines were germinated in ½ MS media and all lines showed 100% germination rates indicating that SMG deletion has not affected the plants’ normal growth and fertility (Supplementary Figure S3).

### Expression analysis of Cas9, SMG and GOI

**FIGURE 4:**
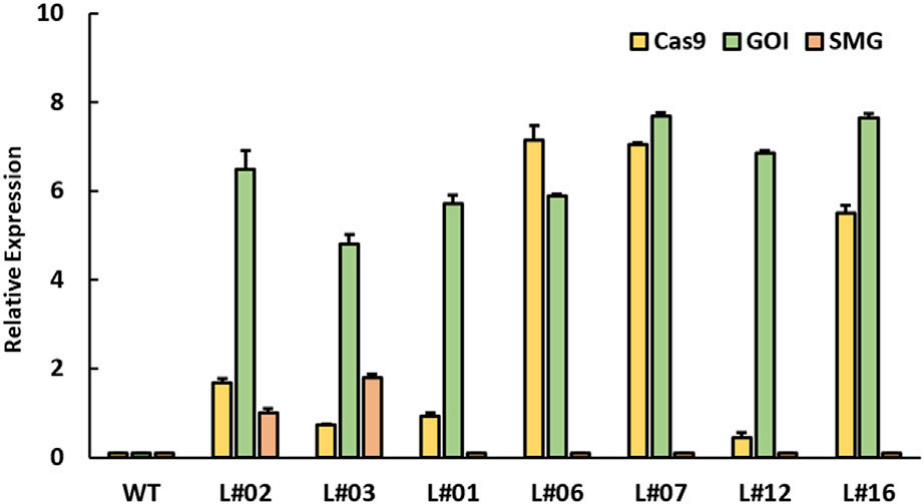
Relative expression of transgenes in pHSE401-SMGDEL transgenic tobacco lines. Quantitative PCR (qPCR) analysis showing the relative expression of three transgenes: cas9, (yellow), aminoglycoside phosphotransferase (GOI, green), and DsRED (SMG, orange) across different transgenic lines. Transgenic tobacco lines L#02, L#03 indicate the SMG undeleted lines, while other bars represent SMG deleted lines (L#01, L#06, L#07, L#12, and L#16). WT: wild-type tobacco plants. qPCR analysis was performed by 2^–ΔΔCT^ method using ACTIN as the reference gene. Samples with Ct ≥ 35 were categorized as undetermined and assigned a value of 0. Error bars represent standard deviation from three biological replicates.

To assess the expression levels of the Cas9 gene, DsRED gene (SMG), and the aminoglycoside phosphotransferase gene (GOI) in our mutated lines, we performed quantitative real-time PCR (qPCR) analyses. We selected two 2.1 kb PCR-positive lines and six SMG-deleted lines for this analysis. As anticipated, the two plants with the 2.1 kb amplicon exhibited increased expression of all three genes compared to wild-type control plants (Figure 4). In contrast, the plants with the 250 bp amplicon expressed both the Cas9 and GOI genes but showed no detectable expression of the DsRED selection marker gene. These findings, combined with the sequencing results, confirm the complete elimination of the SMG cassette in plants with the 250 bp amplicon. This successful removal of the marker gene demonstrates the generation of marker-free transgenic plants.

### Recovery of marker-free, cas9-segregated transgenic plants

T1 seedlings derived from a single SMG-deleted line were genotyped by PCR to assess the presence of the SMG, GOI, and the Cas9 gene, using gene-specific primers. As expected, no SMG cassette was present in all plants analyzed. GOI was retained, and the Cas9 transgene had segregated out in several seedlings (Supplementary Figure S4). These results indicate the successful recovery of marker-free and Cas9-free transgenic plants carrying only the gene of interest.

## Discussion

In this study, we developed and validated a CRISPR-based vector system for precise removal of selectable marker (SMGs) from transgenic plants. CRISPR-based genome editing has proven successful in generating transgene-free mutants in various plant species, as integration of the transgene at genomic loci distinct from the mutation site increases the likelihood of their segregation in the next-generation (Bhattacharjee et al., 2023; Ricroch et al., 2017). Using a multiplexed gRNA strategy, we excised DsRED marker gene from transgenic tobacco plants, while retaining the aminoglycoside phosphotransferase gene (GOI).

Previous studies have shown that using multiple gRNAs significantly increases the likelihood of mutations and large chromosomal deletions (Saito et al., 2024; Song et al., 2017; Han et al., 2014; Song et al., 2016; Ordon et al., 2023). For example, in Arabidopsis, the use of two pairs of gRNAs (four gRNAs in total), each targeting one side of a chromosomal segment, led to a considerably higher deletion frequency (∼2.2 kb) compared to using a single pair of gRNAs (Ordon et al., 2023). Similarly, in soybean, deletion frequencies of 15.6% were achieved for target fragments ranging from 599 to 1,618 bp, and 12.1% for chromosomal segments exceeding 4.5 kb using multiple gRNAs (Cai et al., 2018). We designed four gRNAs, two targeting each side of the SMG cassette, to maximize the chances of successful deletion. While the effects of gRNA orientation on large chromosomal deletions are not fully understood, we opted to design gRNA pairs targeting opposite strands on either side of the SMG cassette based on a study that successfully used multiple gRNAs on opposite strands to generate large chromosomal deletions (Xie et al., 2015). Approximately 10% of the T0 transgenic plants exhibited complete deletion of the SMG cassette, a frequency comparable to the large chromosomal deletions reported in previous studies (Park et al., 2022; Saito et al., 2024; Li et al., 2019).

Traditional gRNA expression cassettes (∼400–500 bp) require separate Pol III promoter and terminator units for each gRNA, which increases plasmid size and complicates delivery. To overcome this, we adopted the polycistronic tRNA-gRNA (PTG) system, where multiple gRNAs are transcribed as a single unit and cleaved by endogenous RNases to release functional gRNAs (Xie and Yang, 2019; Xie et al., 2015; Dong et al., 2017). Following this strategy, we designed a PTG system incorporating the four gRNAs separated by tRNA sequences and expressed it as a single transcript under the Pol III promoter. Our results indicate that the PTG was successfully cleaved into individual gRNAs in pHSE401-SMGDEL transformed plants. When SMG deleted and undeleted plants were sequenced, we observed mutations in targets of all four gRNAs, even though their mutation efficiencies differed. In addition to deletions, insertions and substitutions, we observed inversion of the intervening regions between the two 5′ gRNA targets in one of the lines. Such cas9 mediated inversions, though rarer than indels and substitutions have been previously reported in plants (Zhang et al., 2017; Khosravi et al., 2025). While some studies suggest that the mutation efficiency of gRNAs within a polycistronic tRNA-gRNA (PTG) array decreases with their distance from the Pol III promoter, our findings do not support this trend (Ma et al., 2019). In our study, both 5′ gRNA2 and 3′ gRNA2 exhibited the highest mutation efficiencies, indicating that gRNA position within a PTG does not necessarily correlate with mutation efficiency.

Quantitative real-time PCR (qPCR) analyses showed no detectable expression of marker gene in the SMG-deleted lines, while 2.1 kb PCR-positive lines showed high expression levels of DsRED compared to wild-type control plants. qPCR analysis is widely used for quantifying gene expression levels in plant tissues (Fletcher, 2014). The PCR results combined with qPCR results confirm that no functional copy of SMG is present in the tested SMG-deleted lines.

A couple of recent studies demonstrated the use of a CRISPR/Cas9-based vector to successfully delete unwanted genetic elements from transgenic plants, leaving only the gene of interest (GOI) cassette (Hu et al., 2024; Hu and Yu, 2022). This approach however, involves the simultaneous integration of the transgene followed by the deletion of markers and other elements. A similar approach was demonstrated in rice where CRISPR/Cas9-mediated homology-directed repair (HDR) was utilized to excise unwanted genetic elements including marker genes and cas9 and regions of GOI were recombined to form an intact gene (Tan et al., 2022). In contrast, our approach is specifically designed to delete marker genes from previously transformed and well-characterized transgenic plants.

Similar to ours, a previous report in rice used a dual gRNA system to target either sides of an SMG in a transgenic plant and obtained marker-free plants, but with considerably lower efficiency (Srivastava et al., 2017). Additionally, most plant lines generated displayed abnormal phenotype and complete sterility and no T1 marker-free lines were reported (Srivastava et al., 2017). The authors hypothesized that such high incidence of abnormal phenotype could be due to the deleterious off-target effects. In contrast, none of the 20 transgenic lines analyzed in detail in our study showed abnormalities when grown further. These plants developed, flowered, and set seeds on time, similar to untransformed controls. 100% seed germination was observed when 6 randomly selected complete-deletion lines were analyzed and healthy T1 marker-free transgenic plants were obtained. This highlights the importance of selecting guide RNAs with minimal off-target effects.

Since our design targets regions flanking the SMG cassette in the original vector, the same CRISPR vector can be used to remove SMGs from any transgenic plant transformed with different GOIs as part of the original vector. The only requirement is that the CRISPR vector must carry a different SMG from the original transformation vector. Furthermore, given that an efficient transformation system has already been established for these plants, there is no need to standardize the transformation process for the CRISPR vector. A simple PCR will be sufficient to identify SMG deleted lines in T0 generation. It is possible that the Cas9 T-DNA introduced during re-transformation could integrate into the same chromosome as the original T-DNA containing the GOI. In such cases, tight linkage between the two T-DNAs may hinder effective segregation in the T1 generation, potentially complicating the recovery of Cas9-free, marker-free plants. To minimize this risk, one practical strategy is to screen a larger number of T0 lines to increase the likelihood of identifying individuals with unlinked T-DNA insertions. By starting with the most promising transgenic line intended for commercial release, recovering even a single marker-free transgenic plant would fulfill our objectives.

These factors, along with our design incorporating multiple gRNAs, make our approach highly efficient for developing marker-free transgenic plants. To the best of our knowledge, this is the first report of using the CRISPR-Cas9 system to develop healthy fertile marker-free transgenic plants. Although the gRNA targets were selected with no homology to plant genomic regions, it remains prudent to assess potential off-target effects in any transgenic line intended for commercial use.

## Conclusion

We successfully demonstrated the use of CRISPR/Cas9 technology to eliminate selectable marker genes from transgenic plants. By employing multiple gRNAs targeting both upstream and downstream regions flanking the SMG cassette, we achieved a 10% complete deletion efficiency in the T0 generation. Molecular analyses, including PCR, sequencing, and qPCR, confirmed the precise deletion of the marker gene without impacting the gene of interest or plant development. SMG-free plants exhibited normal growth, flowering, and seed production, highlighting the utility and reliability of this approach. This strategy provides a simple and effective solution for eliminating selection marker genes from existing transgenic plants, offering significant advantages for regulatory compliance and public acceptance of genetically modified plants.

## Key message

A multiplex CRISPR/Cas9 system was employed to efficiently remove selectable marker genes (SMGs) from transgenic plants. This approach enables precise deletion of SMGs, ensuring marker-free transgenic crops for improved commercialization and regulatory approval.

## Data Availability

The datasets generated and analyzed during this study are available on Figshare: qRT-PCR data at DOI: 10.6084/m9.figshare.29965538 and CRISPR mutation-sequencing data at DOI: 10.6084/m9.figshare.29965547. All other relevant data are included in this article and its Supplementary Material; further inquiries can be directed to the corresponding author.
