# Supplementary material for: Application of a multiplex CRISPR/Cas9 strategy for elimination of selection markers from transgenic plants
Source: Front Genome Ed. 2025 Sep 3;7:1633104. doi: 10.3389/fgeed.2025.1633104 (PMC12440875; doi:10.3389/fgeed.2025.1633104)
Supplement: Supplementary file 1 [file DataSheet1.pdf]

Table S1. Primers used in this study.

Primer name	Sequence (5'-3')
RED_FRAG_F	TCACTGTTGATACATATGGCCTCCTCCGAGAAC
RED_FRAG_R	TTCAGAATTGTCGACCTACAGGAACAGGTGGTGG
Gene1_SEQ_F	CACGGGGGACTCTAGATACATCAC
Gene1_SEQ_R	CTTAAGCACACAAGCTAGCTTTTTATTTGACAC
5' gRNA1_F	GTTAGAGAGGCTTACGCAGCGTTTTAGAGCTAGAAATAGCAAGTTAAAATAAGGC
5' gRNA1_R	TTTGTTGCACCGACTCGGTGCC
5' gRNA2_F	CACCGAGTCGGTGCAACAAAGCACCAGTGGTCTAGTGGTAGAA
5' gRNA2_R	TCTAAAACGGAGATTATTGCTCGGGTAGTGCACCAGCCGGGAA
3' gRNA1_F	CTACCCGAGCAATAATCTCCGTTTTAGAGCTAGAAATAGCAAGTTAAAATAAGGC
3' gRNA1_R	CCTGGATCCCGGGAATTCGTATGCACCAGCCGGGAA
3' gRNA2_F	TACGAATTCCCGGGATCCAGGTTTTAGAGCTAGAAATAGCAAGTTAAAATAAGGC
3' gRNA2_R	GCTCTAAAACGGGAATTCGTAATCATGGTCTGCACCAGCCGGGAA
AT_U6_F	GAAGAGAAGCAGGCCCATTT
AT_U6_R	CCCCAGAAATTGAACGCCG
KAN_F	GATGGATTGCACGCAGGTTC
KAN_R	TCATTTCGAACCCCAGAGTCC
HYG_F	GTGCTTGACATTGGGGAGTT
HYG_R	GGTTTCCACTATCGGCGAG
pRED-AN_F	ACAATCTGATCCTGGCGAAAG
pRED-AN_R	TGTGTGGAATTGTGAGCGGATAAC
RED_RT_F	GCCACTACCTGGTGGAGTTC
RED_RT_R	GGTGTAGTCCTCGTTGTGGG
KAN_RT_F	GATGGATTGCACGCAGGTTC
KAN_RT_R	CAGCCGATTGTCTGTTGTGC
CAS_RT_F	CATTAATGCGTCAGGCGTCG
CAS_RT_R	GCGCAATGAGATTCCCGAAC
NtACT9_RT_F	CCTGAGGTCCTTTTCCAACCA
NtACT9_RT_R	GGATTCCGGCAGCTTCCATT

Figure S1: Vector maps

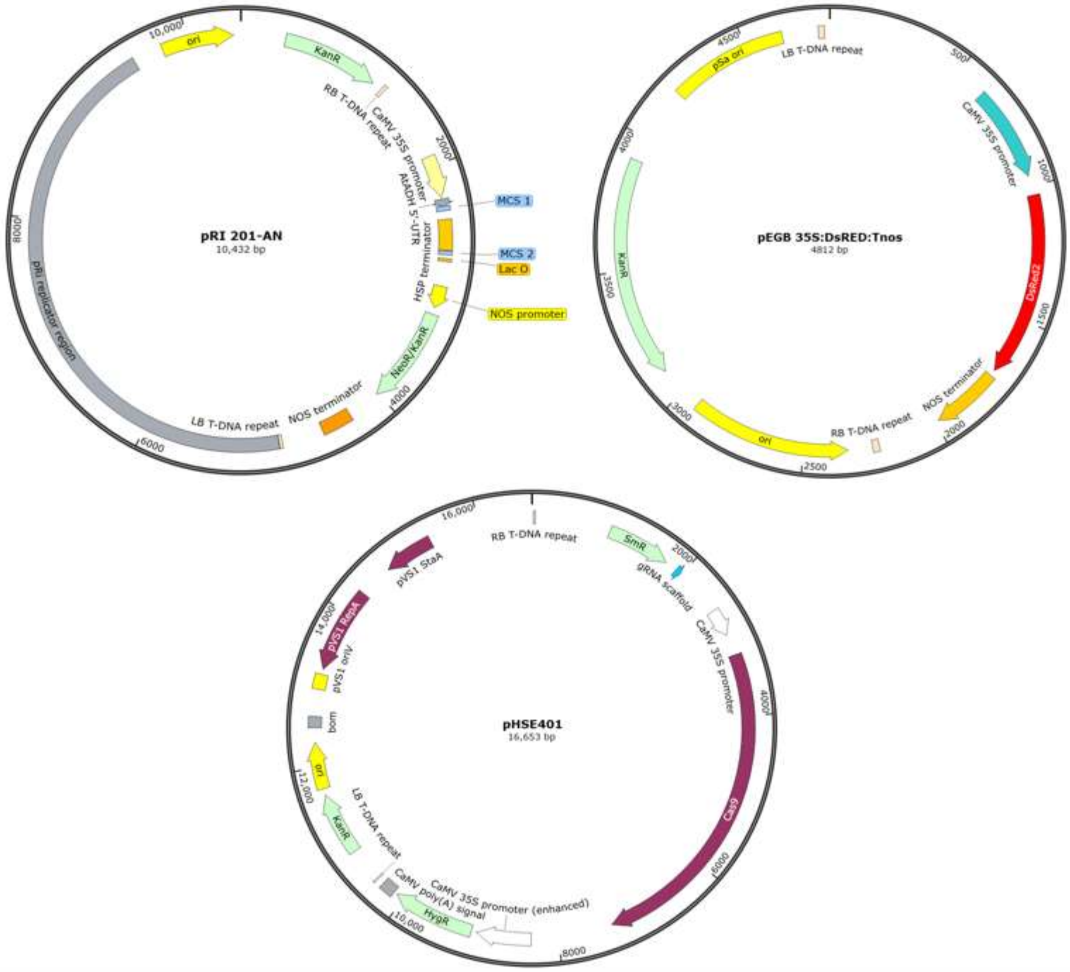

Figure S2: PTG used in this study

5'GTTAGAGAGGCTTACGCAGCGTTTTAGAGCTAGAAATAGCAAGTTAAAATAAGGCTAGTCCGTTATCAACTTGA
AAAAGTGGCACCGAGTCGGTGCAACAAAGCACCAGTGGTCTAGTGGTAGAATAGTACCCTGCCACGGTACAGACC
CGGGTTCGATTCCCGGCTGGTGCACTACCCGAGCAATAATCTCCGTTTTAGAGCTAGAAATAGCAAGTTAAAATAA
GGCTAGTCCGTTATCAACTTGAAAAAGTGGCACCGAGTCGGTGCAACAAAGCACCAGTGGTCTAGTGGTAGAATA
GTACCCTGCCACGGTACAGACCCGGGTTCGATTCCCGGCTGGTGCATACGAATTCCCGGGATCCAGGTTTTAGAGC
TAGAAATAGCAAGTTAAAATAAGGCTAGTCCGTTATCAACTTGAAAAAGTGGCACCGAGTCGGTGCAACAAAGCA
CCAGTGGTCTAGTGGTAGAATAGTACCCTGCCACGGTACAGACCCGGGTTCGATTCCCGGCTGGTGCAGACCATG
ATTACGAATTCCCGTTTTAGAGCTAGAAATAGCAAGTTAAAATAAGGCTAGTCCGTTATCAACTTGAAAAAGTGGC
ACCGAGTCGGTGC-3'

gRNAs, gRNA scaffold, tRNA

Figure S3

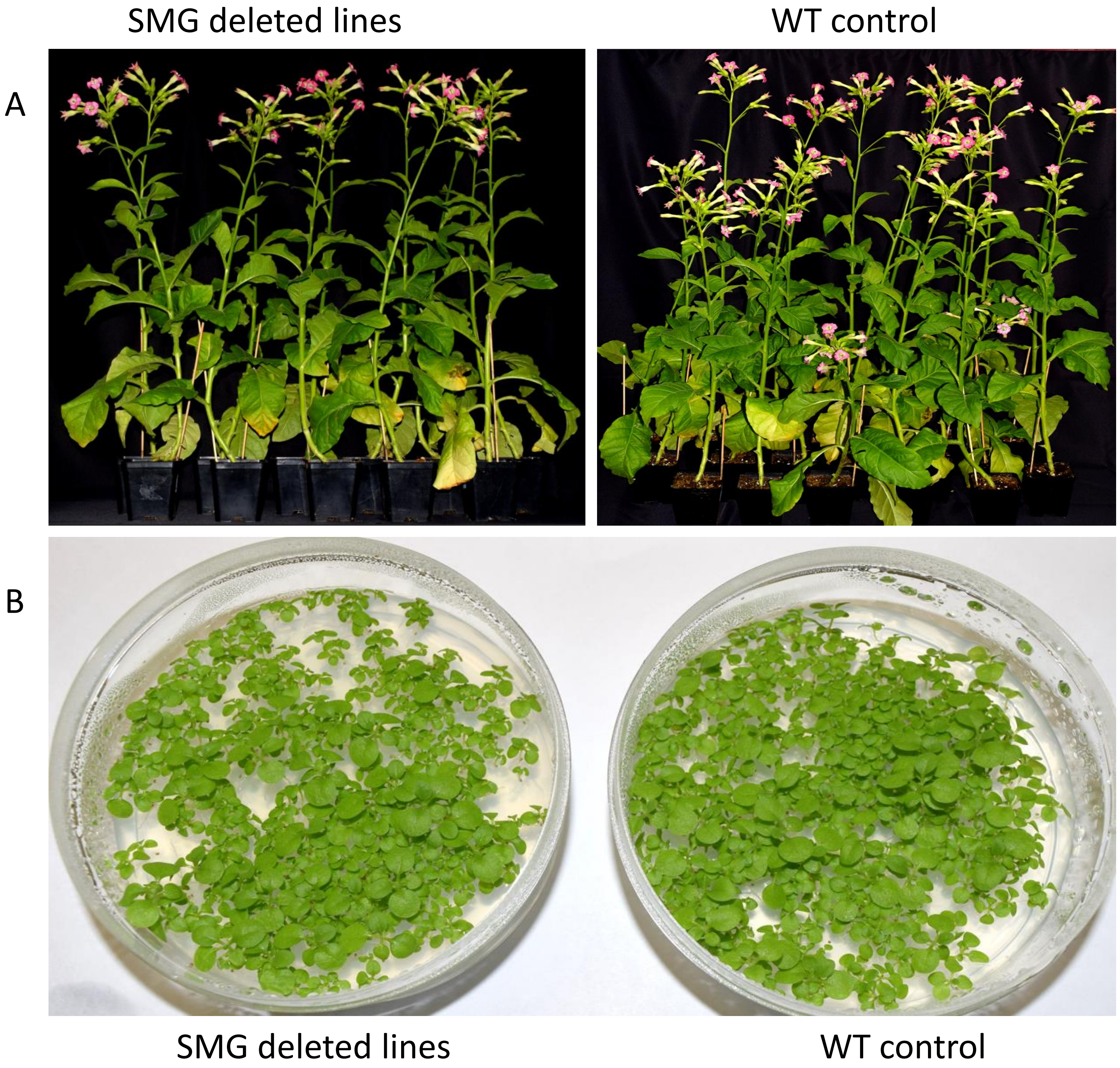

Growth of SMG deleted lines in comparison with wild type control tobacco plants. A) T0 lines at flowering stage, B) T0 seed germination

Figure S4

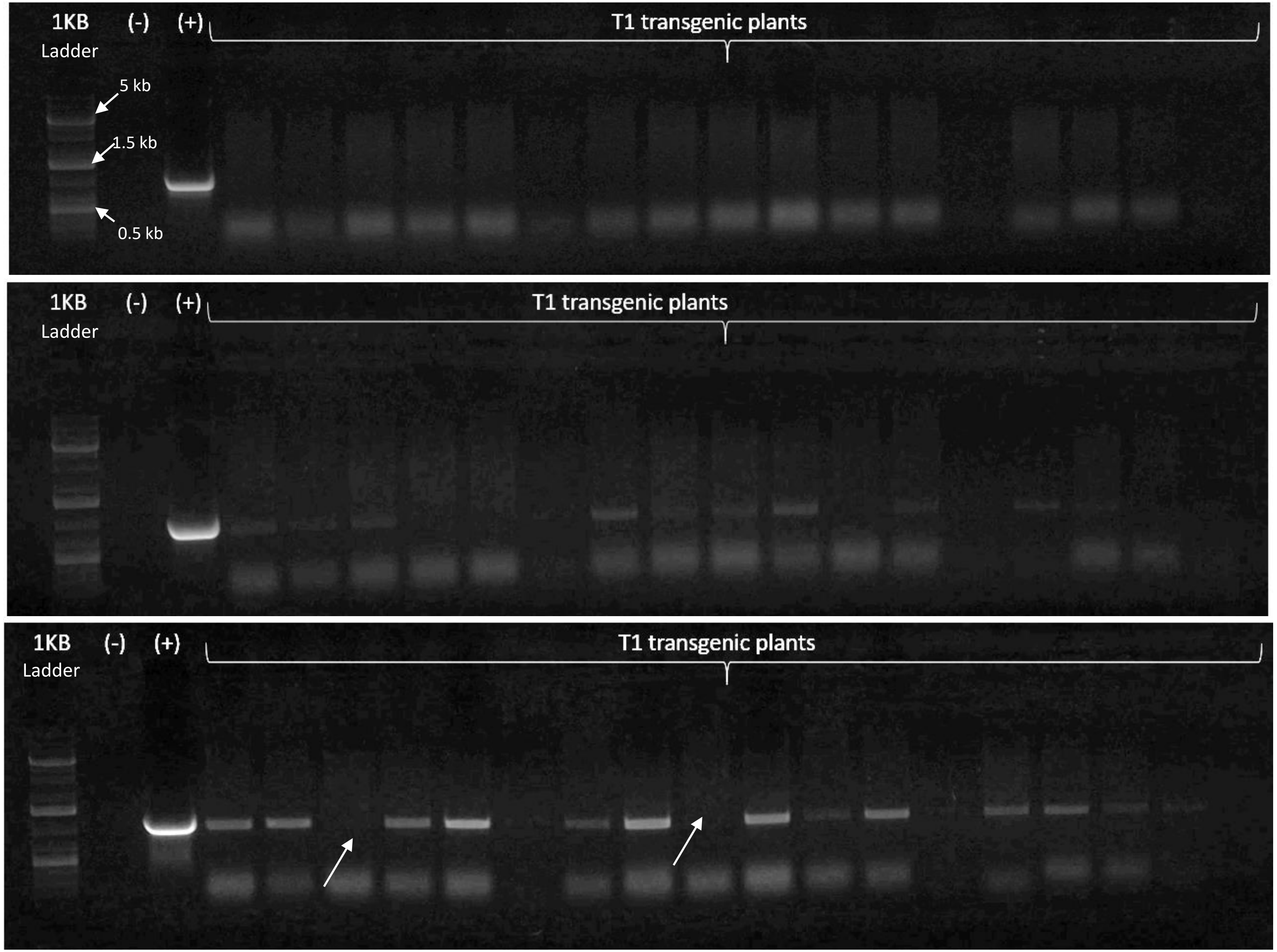

Figure S4: Genotyping of T1 seedlings derived from a single SMG-deleted line. A) PCR gor SMG, B) PCR for GOI, C) PCR for Cas9 gene. (-) – negative control (wild-type plant DNA), (+) – positive control (respective plasmid). Arrows indicate marker-free, cas9-segregated transgenic plants
